# Knowledge, Perceived Risk and Utilization of Prostate Cancer Screening Services among Men in Dar Es Salaam, Tanzania

**DOI:** 10.1155/2019/2463048

**Published:** 2019-12-03

**Authors:** Fidelis Charles Bugoye, Germana Henry Leyna, Kåre Moen, Elia John Mmbaga

**Affiliations:** ^1^Department of Forensic Science and DNA Services, Government Chemist Laboratory Authority, Dar es Salaam, Tanzania; ^2^Department of Epidemiology and Biostatistics, Muhimbili University of Health and Allied Sciences, Dar es Salaam, Tanzania; ^3^Department of Community Medicine and Global Health, University of Oslo, Norway

## Abstract

**Background:**

Late diagnosis of prostate cancer is common in low and middle income countries and contributes to high morbidity and mortality of the disease. Utilization of prostate cancer screening services plays a major role in prevention of adverse outcomes. However, there is limited information on the knowledge about, the perceived risk of, and the utilization of prostate cancer screening in Tanzania.

**Objective:**

To determine knowledge and perceived risk of prostate cancer, and the utilization of prostate cancer screening services, and associated factors, among men in Dar es Salaam, Tanzania.

**Design:**

A population-based cross-sectional study involving men aged 40 years and above living in Dar es Salaam was conducted between May and August, 2018.

**Methodology:**

Participants were recruited through multistage random sampling and took part in structured face-to-face interviews. Categorical variables were summarized using proportions while continuous variables were summarized as medians and inter-quarterly range (IQR). Chi square test was used to compare differences between proportions, and logistic regression modelling was used to determine factors associated with utilization of prostate cancer screening. Both crude and adjusted odds ratios (OR), with corresponding 95% confidence intervals, are reported. All analyses were two-tailed and the significance level set at 5%.

**Results:**

A total of 388 men with a median age of 53 years (IQR 44–55) participated. Half (52.1%) had poor knowledge about prostate cancer and prostate cancer screening. A third (32.3%, *n* = 125) perceived the risk of prostate cancer to be low. Only 30 respondents (7.7%) had ever been screened for prostate cancer. Utilization of prostate cancer screening services was independently associated with age above 60 years [AOR = 21.46, 95% CI: 6.23, 73.93], monthly income above 305 US Dollars [AOR = 15.68, 95% CI: 4.60, 53.48], the perceived risk of prostate cancer [AOR = 16.34, 95% CI: 7.82, 14.92] and knowledge about prostate cancer [AOR = 67.71, 95% CI: 8.20, 559.57].

**Conclusions:**

Knowledge about prostate cancer and prostate cancer screening services was low among men in Dar es Salaam with a third perceiving themselves to be at no risk for the disease. Utilization of screening services was low and associated with low income, younger age, low perceived risk of prostate cancer and low knowledge about the disease. Intervention measures aiming to increase knowledge about prostate cancer and screening services, and affordable provision of services, are urgently called for.

## 1. Introduction

Breast cancer in women and prostate cancer in men have now become the most commonly diagnosed cancers in many Sub-Saharan African countries. Prostate cancer is an adenocarcinoma of the male prostate and is one of the most common cancers in the world and a leading cause of cancer related deaths among men globally [1–3]. While prostate cancer is thus an important health burden among men across the globe, the highest incidence rates are found in sub-Saharan Africa [1, 2, 4, 5]. A systematic review of prostate cancer in Africa estimated an overall continent-wide pooled incidence rate of 21.95/100,000 population [2]. The Tanzania Cancer Registry indicates that prostate cancer is the most common cancer among men in Tanzania (followed by Karposi's sarcoma) with an incidence of 3,434 cases per year in 2012 [6].

While screening for prostate cancer using the prostate-specific antigen blood test (PSA) and digital rectal examination (DRE) are effective available measures for early detection of disease, utilization of these services ranges from 0% to 15% in Africa [4, 7–10]. A majority of patients with prostate cancer in Tanzania report to health facilities when the cancer is locally advanced or metastatic. A recent study reported that 21% of men attending routine transurethral prostatectomy were incidentally identified to have prostate carcinoma [11].

Various factors associated with utilization of prostate cancer screening have been documented both in developed and developing countries. A recent study reported that African and African American men were less likely than European and European American men to seek prostate cancer-screening as a direct or indirect consequence of financial barriers, lack of health insurance, and/or poor health-seeking behaviour [10]. Studies have also found that perceived risk of prostate cancer and low knowledge about the disease and prostate cancer screening methods play an important role in cancer screening utilization [7, 10, 12–15]. Good knowledge about and understanding of a disease is generally associated with a more optimal healthcare-seeking attitude and behaviour [9, 13, 16].

Recently there have been a number of public campaigns to promote prostate cancer awareness in Tanzania, but the utilization of prostate cancer screening services has not been evaluated. The present study therefore aimed to examine the level of knowledge about prostate cancer, perceived risk of the disease, and utilization of prostate cancer screening services, and their associated factors, among men in Dar es Salaam in order to inform ongoing and new intervention measures.

## 2. Population and Methods

### 2.1. Study Design and Population

A population-based cross-sectional study was conducted from May to August 2018 among men aged 40 years and above who were residents of Dar es Salaam (had an address in the city and had lived in it for more than one year). Based on the level of knowledge of prostate cancer and prostate cancer screening reported in a study from Kenya [17], sample size calculation indicated that recruitment of 384 study participants would yield 80% power at 5% error rate to estimate our outcome.

### 2.2. Sampling Procedure

Multistage cluster sampling was used to recruit participants for this study. We first randomly selected two of Dar es Salaam's five administrative municipalities (Kinondoni and Ubungo). From each of these, two wards were selected randomly (Sinza, Goba, Ndugumbi and Mwananyamala), from each of which two streets were randomly selected, from each of which two administrative cells were randomly selected. A list of households with eligible men was obtained from the ward office and served as our sampling frame within these administrative cells. Eligible participants were recruited through house to house visit during the evening.

### 2.3. Data Collection Tool

A questionnaire was followed during structured face-to-face interviews to collect data on socio-demographic characteristics of the respondents, their knowledge about and perceived risk of prostate cancer, and their utilization of screening services. A set of questions were used to measure knowledge of prostate cancer and pretested to assess their face validity prior to actual data collection. All questions were developed in English and then translated into Swahili, the language used during the interview.

### 2.4. Data Analysis

Data were entered into and analysed in the Statistical Packages for the Social Sciences (SPSS) version 20.0. Categorical variables were summarized using proportions while continuous variables were summarized as medians and inter-quarterly range (IQR). Knowledge (8 questions) and internal consistency was examined using Cronbach's alpha (0.856). Chi square test was used to compare differences between proportions and both bivariate and multivariate logistic regression modelling was used to determine independent factors influencing the utilization of prostate cancer screening services. Both crude odds ratios (OR) and adjusted odds ratios (AOR) were calculated and presented with corresponding 95% confidence intervals. All analyses were two-tailed and the significance level set at 5%.

### 2.5. Ethical Issues

Ethical approval for this research was obtained from MUHAS Ethical Research Committee (MU/PGS/SAEC/VOL.IX/56) and the permission to conduct the study was thereafter requested from local authorities in Kinondoni and Ubungo municipalities. All study participants provided written informed consent prior to the interview. Confidentiality was maintained and no names were recorded in any questionnaire. Interviews were conducted in privacy.

## 3. Results

About 400 men were approached to consent and participate in the study-a total of 388 (97%) men with a median age of 53 years (IQR: 44–55) living in Ubungo and Kindondoni municipality of Dar es Salaam participated in the study. Majority were married (85.1%) and about a third had completed secondary education with only 1% reporting no formal education. Nearly half (45.6%) were employed whereas 39.9% engaged in business and 10.8% were retired. The median monthly earning among respondents was 305 US Dollars (IQR = 174–348 US Dollars) (Table 1).

### 3.1. Knowledge about Prostate Cancer and Prostate Cancer Screening Methods

**Figure 1:**
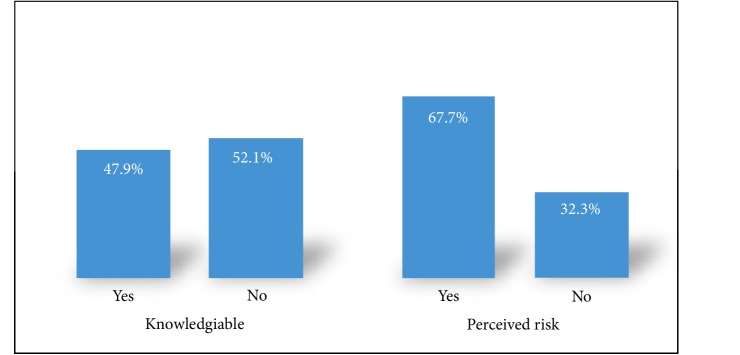
Level of knowledge and perceived risk of prostate cancer.

Overall, 48% (*n* = 186) of the study participants were knowledgable about prostate cancer and prostate cancer screening method (Figure 1).

Table 2 presents the proportion of responses to various knowledge questions. Knowing someone previously affected by prostate cancer, was reported by about half of the interviewees (46.1%, *n* = 179) while knowing someone currently was reported by 17.3% (*n* = 67). The majority of participants (93.3%, *n* = 362) knew that prostate cancer is a disease that affects men, nearly two thirds (63.7%, *n* = 247) knew that the disease is severe, and half (51.3%, *n* = 199) knew that the disease is curable if detected early. While only 27.3% (*n* = 106) had knowledge about the symptoms of prostate cancer, 62.4% (*n* = 242) knew about the screening methods (PSA and/or DRE).

### 3.2. Participants Perceived Risk of Prostate Cancer

When participants were asked how they perceived the risk of prostate cancer to be among men in general, 24.6% (*n* = 95) said men are at high risk, 62% (*n* = 240) said they are at some risk and 23.4% (*n* = 52) that they are at no risk at all. With regards to the interviewee's individual (personal) risk of prostate cancer, 13.7% (*n* = 53) felt they were at high risk, half (54.0%, *n* = 209) thought they were at some risk whereas about a third (32.3%, *n* = 125) assumed that they were at no risk of developing prostate cancer (Figure 1).

### 3.3. Utilization of Prostate Cancer Screening Services among Respondents

The proportion of study participants who had ever utilized prostate cancer screening services was only 7.7% (*n* = 30). Participants who were above 60 years, who had a monthly income above 305 US Dollars, who were knowledgable about prostate cancer and who perceived themselves to be at risk of prostate cancer, were more likely to have utilized screening services in the bivariate analysis (Table 3).

### 3.4. Independent Factors Associated with Utilization of Prostate Cancer Screening Services

Results from the bivariate and multivariate logistic regression modelling of independent factors associated with utilization of screening services are presented in Table 3. Being above 60 years of age was associated with a 21 times (AOR = 21.46; 95% CI: 6.23, 73.93) higher odd of utilizing a screening method in this population. Participants who had a monthly income above 305 US Dollars were almost 16 times (AOR = 15.68; 95% CI: 4.60, 53.48) more likely to report utilization of screening services for prostate cancer than those with lower income. Being knowledgable about prostate cancer and prostate cancer screening methods (AOR = 67.72; 95% CI: 8.20, 559.57) and perceived risk for the development of prostate cancer (AOR = 16.34; 95% CI: 7.82, 214.92) were also independently associated with higher odds of utilizing screening services among participants in this study (Table 3).

## 4. Discussion

This study aimed to estimate the level of knowledge about prostate cancer and prostate cancer screening services, perceived risk of prostate cancer, utilization of prostate cancer screening services, and associated factors, among men aged 40 years and above in Dar es Salaam, Tanzania.

Our findings indicate that half of the men in this population (52%) have poor knowledge about prostate cancer. Similar findings have recently been reported among men in Nigeria, South Africa and black men in the Caribbean, America and Africa [10, 13, 14, 18, 19]. Our study findings also corroborate with similar study conducted in Ghana which support the assertation that poor knowledge about prostate cancer is associated with low utilization of screening services [31]. Despite ongoing efforts to raise prostate cancer awareness and knowledge in many African countries, it is obvious that more needs to be done to ensure that men understand the disease and take appropriate action.

Perceived risk of disease has been associated with intention to seek health services including cancer screening services [13, 15]. A third of participants in this study (32%) perceived themselves to not be at risk of prostate cancer, similar to what has been reported in nearby East African country of Kenya and elsewhere in Africa [5, 20]. A study on the factors influencing men's decision regarding prostate cancer screening in the United States and Africa reported perceived risk of developing prostate cancer to be an important factor for screening uptake [21]. Utilization of health services has been reported to strongly correlate with good knowledge of disease, perceived risk and knowledge about the disease consequencies [22]. Scaling up ongoing efforts to educate the community will go a long way in improving risk perception hence promoting health seeking behaviours.

Utilization of screening services for prostate cancer was uncommon among participants in this study. Only 8% of men aged 40 years and above, reported to have ever been screened for prostate cancer. Studies in Nigeria, Kenya and elsewhere in Africa have also found alarmingly low use of screening services for prostate cancer. Low utilization of prostate cancer screening have been associated with poor knowledge about prostate cancer and the screening methods in black populations in America and Africa [10, 15, 19, 23, 24]. It is well known that low utilization of prostate screening services contributes to late diagnosis and increased mortality and morbidity related to prostate cancer [1, 4, 5, 25]. Overscreening has potential to cause harm through overdiagnosis and unnessesary invasive procedures, hence its critical that men should be subjected to physical screening based on risk age and having suggestive symptoms. Given that early diagnosis serves life, these findings underscore the importance of efforts geared towards sensitization and knowledge creation among men on the benefits of prostate cancer screening and screening methods in developing countries.

In this study, low utilization of prostate cancer screening services were associated with age, monthly income, and knowledge about prostate cancer and screening services. The association between service utilization and older age may be a result of prolonged exposure to awareness campaigns on prostate cancer but also increased risk and symptoms of prostate cancer with age that may prompt health seeking. Old age has also been associated with decreased sexual activity and increased risk of prostate cancer hence seeking screening services [26]. Similar findings have also been reported elsewhere in Africa, Asia and beyond [3, 8, 15, 23, 27].

Higher income was associated with increased utilization of prostate cancer screening services in this study. Access to health services including screening for cancer has been associated with socio-economic status in several studies worldwide. Education level and income are highly correlated and people who are educated not only have higher odds of better income but also can have access to health education messages, internalize them and act on them. Moreover, income has also been associated with access to health services through increased ability to pay in areas with low coverage of health insurance like Tanzania [28–30]. Though, 48% of men were found to be aware of the diseases, the utilization of screening was considerably low (8%). This low utilization of screening services could be explained by low perceived risk, fear of being positively diagnosed, beliefs and cultural barriers towards the screening methods including Digital Rectal Examination (DRE) which is considered to be embarrassing, painful and uncomfortable among men as reported elsewhere in African countries [32–34].

As described earlier, perceived disease risk and knowledge are strong predictors of access to health services. In this study, we found a strong association between the perceived risk of prostate cancer and knowledge of prostate cancer and screening services with utilization of services. It is therefore of paramount importance to scale up ongoing prostate cancer campaigns among males above 40 years in the country.

The findings presented here should be interpreted in light of a number of limitations. Firstly, the cross sectional nature of the design limits the ability to make causal inference for factors identified to be associated with prostate cancer screening. However, most factors described have also been published in other studies utilizing various designs and have consistently been associated with service utilization. Secondly, the sample size was calculated to estimate awareness of the screening method and its power to estimate relatively low rate of use is limited. Therefore our estimates of utilization of prostate cancer screening services should be interpreted with caution; thirdly, screening for prostate cancer involves a sensitive procedure particulary if DRE was used. This may have resulted in underestimation of our proportion of utilization due to desirability bias. Efforts were made to build good rapport during interviews and we believe that the accuracy of our estimates may have been strengthened by this, given that these estimates were similar to estimates from elsewhere in Africa.

## 5. Conclusions and Recommendations

This study revealed that the level of knowledge about prostate cancer and screening services is alarmingly low among men in Dar es Salaam. Utilization of screening services for prostate cancer are rare in this population and associated with poor perceived disease risk, low income, lack of knowledge and younger age. Intervention measures aiming at increasing knowledge about prostate cancer and screening services need to be scaled up alongside ensuring that screening services are freely available.

## Figures and Tables

**Table 1:** Distribution of socio-economic and demographic characteristics of the study respondents (*n* = 388).

Variable	*n* (%)
Median age (IQR)	53 (44; 55)

*Area of residence*	
Kinondoni	194 (50.0)
Ubungo	194 (50.0)

*Marital status*	
Married	330 (85.1)
Separated	14 (3.6)
Widowed	17 (4.4)
Single	27 (7.0)

*Level of education*	
No formal education	12 (3.1)
Primary education	103 (26.5)
Secondary education	129 (33.2)
Diploma	54 (13.9)
University	90 (23.2)

*Current occupation*	
Employed	177 (45.6)
Business	155 (39.9)
Retired	42 (10.8)
Not working	5 (1.3)
Agriculture	9 (2.3)
*Median monthly earning in tzs (IQR)*	305 (174–348) US Dollars

**Table 2:** Participants' knowledge about prostate cancer and screening methods (*n* = 388).

Variables	*n*	%
Know someone who has had prostate cancer before		
Yes	179	46.1
No	209	53.9
Know that prostate cancer is a deadly disease		
Yes	247	63.7
No	141	36.3
Know someone who is suffering from prostate cancer today		
Yes	67	17.3
No	321	82.7
Know persons of which sex that are affected by prostate cancer		
Yes	362	93.3
No	26	6.7
Know factors that could make a person more likely to develop prostate cancer		
Yes	153	39.4
No	235	60.6
Familiar with symptoms of prostate cancer		
Yes	106	27.3
No	282	72.7
Prostate cancer curable		
Yes	199	51.3
No	189	48.7
Know prostate cancer screening methods		
Yes	242	62.4
No	146	37.6

**Table 3:** Univariate and multivariate logistic regression of factors associated with utilization of prostate cancer screening services.

Variable		Total	Screened *n* (%)	Univariate			Multivariate		
				COR	95% CI	*p*-value	AOR	95% CI	*p*-value
Age	40–50	179	5 (2.8)	1			1		
	51–60	131	5 (3.8)	1.38	(0.39–4.87)	0.616	2.13	(0.55–8.22)	0.274
	Above 60	78	20 (25.6)	12.00	(4.3–33.41)	<0.001	21.46	(6.23–73.93)	<0.001
Marital status	Married	330	24 (7.3)	0.68	(0.27–1.74)	0.422			
	Not married	58	6 (10.3)	1					
Education	≤Primary	115	6 (5.2)	0.61	(0.22–1.67)	0.331			
	Secondary	129	12 (9.3)	1.13	(0.49–2.61)	0.778			
	College/University	144	12 (8.3)	1			1		
Income	>305USD	147	26 (17.7)	12.52	(4.27–36.67)	<0.001	15.68	(4.60–53.48)	<0.001
	≤305USD	237	4 (1.7)	1			1		
Knowledge	Good	186	29 (15.6)	37.13	(5.00–275.54)	<0.001	67.72	(8.20–559.57)	<0.001
	Poor	202	1 (0.5)	1			1		
Perceived risk	Yes	263	34(12.9)	13.4	(6.12–187.71)	<0.001	16.34	(7.82–214.92)	<0.001
	No	125	2(1.6)	1			1		

## Data Availability

The data used to support the findings of this study are included within the article.
